# Containment of a KPC-CRE Outbreak Associated with Premise Plumbing in a Long-Term Care Facility— Minnesota, 2022-2023

**DOI:** 10.1017/ash.2024.289

**Published:** 2024-09-16

**Authors:** Laura Tourdot, Jennifer Dale, Christine Lees, Bradley Craft, John Kaiyalethe, Paula Snippes Vagnone, Sarah Lim, Krista Knowles, Tammy Hale, Kristi Juaire, Jacy Walters, Ruth Lynfield

**Affiliations:** Minnesota Department of Health; MN Dept. of Health, Public Health Lab

## Abstract

**Background:** On March 23, 2022, the Minnesota Department of Health (MDH) was notified of Klebsiella pneumoniae carbapenemase (KPC)-producing Klebsiella oxytoca isolated from a resident’s urine in long-term care facility A (LTCF-A). Carbapenem-resistant Enterobacterales (CRE) are reportable statewide with required isolate submission to MDH Public Health Laboratory (MDH-PHL), where carbapenemase production and mechanism identification is confirmed. **Methods:** MDH partnered with LTCF-A on a containment response, including infection prevention and control (IPC) measures, KPC-CRE education, and colonization screening. Rectal swabs were screened for carbapenemase genes by real-time PCR (Cepheid Xpert Carba-R), with positive specimens undergoing culture, isolation, and whole genome sequencing (WGS). MDH-PHL conducted WGS including multilocus sequence typing (MLST) and single nucleotide polymorphism (SNP) analysis to describe genetic relationships among isolates. When screening indicated a potential environmental source, due to species diversity and ongoing resident transmission, an environmental screening plan was developed including collection of premise plumbing samples from room faucets, aerators, sinks, toilets, and shared shower drains. **Results:** KPC-CRE was detected in 23 residents (urine, n=2; rectal swab, n=21) during March 2022–November 2023. 21 isolates comprising 10 Enterobacterales species were cultured from KPC-positive screening specimens. SNP analysis performed on bacteria of the same species demonstrated 5 distinct clusters of relatedness comprising 2-3 residents per cluster (Cluster 1: Klebsiella oxytoca, n=3; Cluster 2: Klebsiella oxytoca, n=3; Cluster 3: Escherichia coli, n=2; Cluster 4: Klebsiella pneumoniae, n=2; Cluster 5: Raoultella planticola, n=2). 7 KPC-positive resident specimens did not yield a culturable organism. KPC-CRE was detected throughout the premise plumbing including 8 of 9 shared shower room drains and 6 of 75 resident room sink drains. WGS and SNP analysis suggest relatedness among resident and environmental KPC-CRE isolates. Gaps in IPC measures including hand hygiene, use of personal protective equipment (PPE), environmental cleaning and disinfection, and sink hygiene practices were observed during onsite assessments. Use of an EPA-registered biofilm disinfectant in facility drains and repeated environmental sampling has demonstrated a decrease in KPC-harboring bacteria within the premise plumbing, but not complete elimination. **Conclusion:** Containing the spread of KPC-CRE within LTCF-A has been challenging due to environmental reservoirs of KPC-CRE along with insufficient implementation of IPC practices. Continued colonization screening has been necessary to detect newly colonized residents and reinforce efforts to increase IPC compliance. Strict implementation and adherence to IPC measures, including those that minimize the spread of KPC-CRE from facility premise plumbing, are needed to fully halt KPC-CRE transmission within LTCF-A.

